# Supplementary material for: A yeast phenomic model for the influence of Warburg metabolism on genetic buffering of doxorubicin
Source: Cancer Metab. 2019 Oct 23;7:9. doi: 10.1186/s40170-019-0201-3 (PMC6806529; doi:10.1186/s40170-019-0201-3)
Supplement: Supplementary file 9 — Additional file 9. GO term-specific heatmaps for REMc/GTF-enriched clusters. GO term-specific heatmaps for significant GO process terms were generated as described in methods and Figs. 3 and 4. Any related child terms are presented in subsequent pages of the parent file name. GO terms with more than 100 children, with 2 or fewer genes annotated to the term, or a file size over 300KB are not shown. All heatmaps are generated with the same layout (see Figs. 3 and 4). [file 40170_2019_201_MOESM9_ESM.bz2 › Additional_File9_GOTermHeatmaps/Additional_File9_GOTermHeatmap/ribonucleoprotein_complex_assembly.pdf]

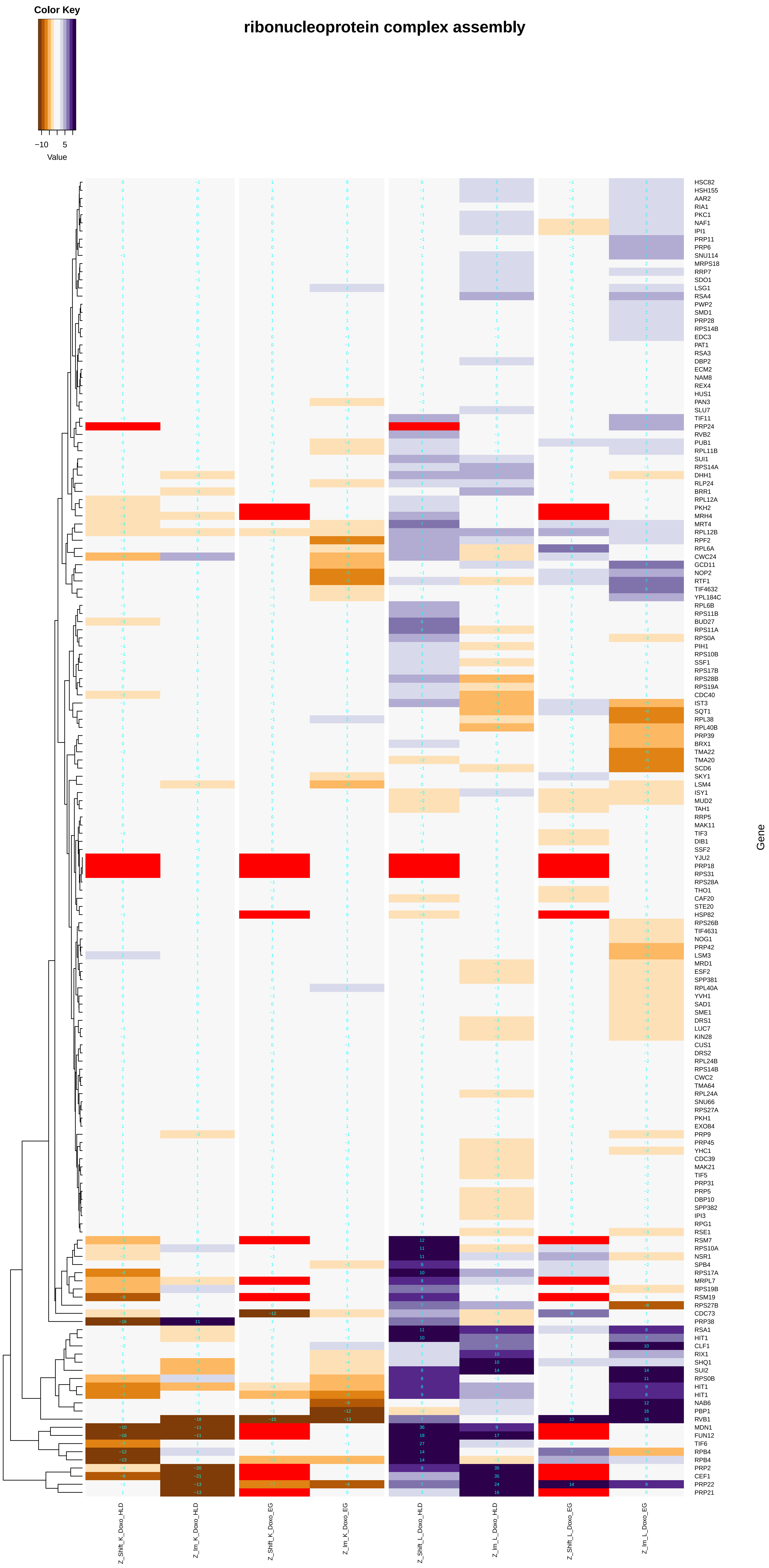
ribonucleoprotein complex assembly
Color Key
−10
5
Value
Gene
Z_Shift_K_Doxo_HLD
Z_Im_K_Doxo_HLD
Z_Shift_K_Doxo_EG
Z_Im_K_Doxo_EG
Z_Shift_L_Doxo_HLD
Z_Im_L_Doxo_HLD
Z_Shift_L_Doxo_EG
Z_Im_L_Doxo_EG

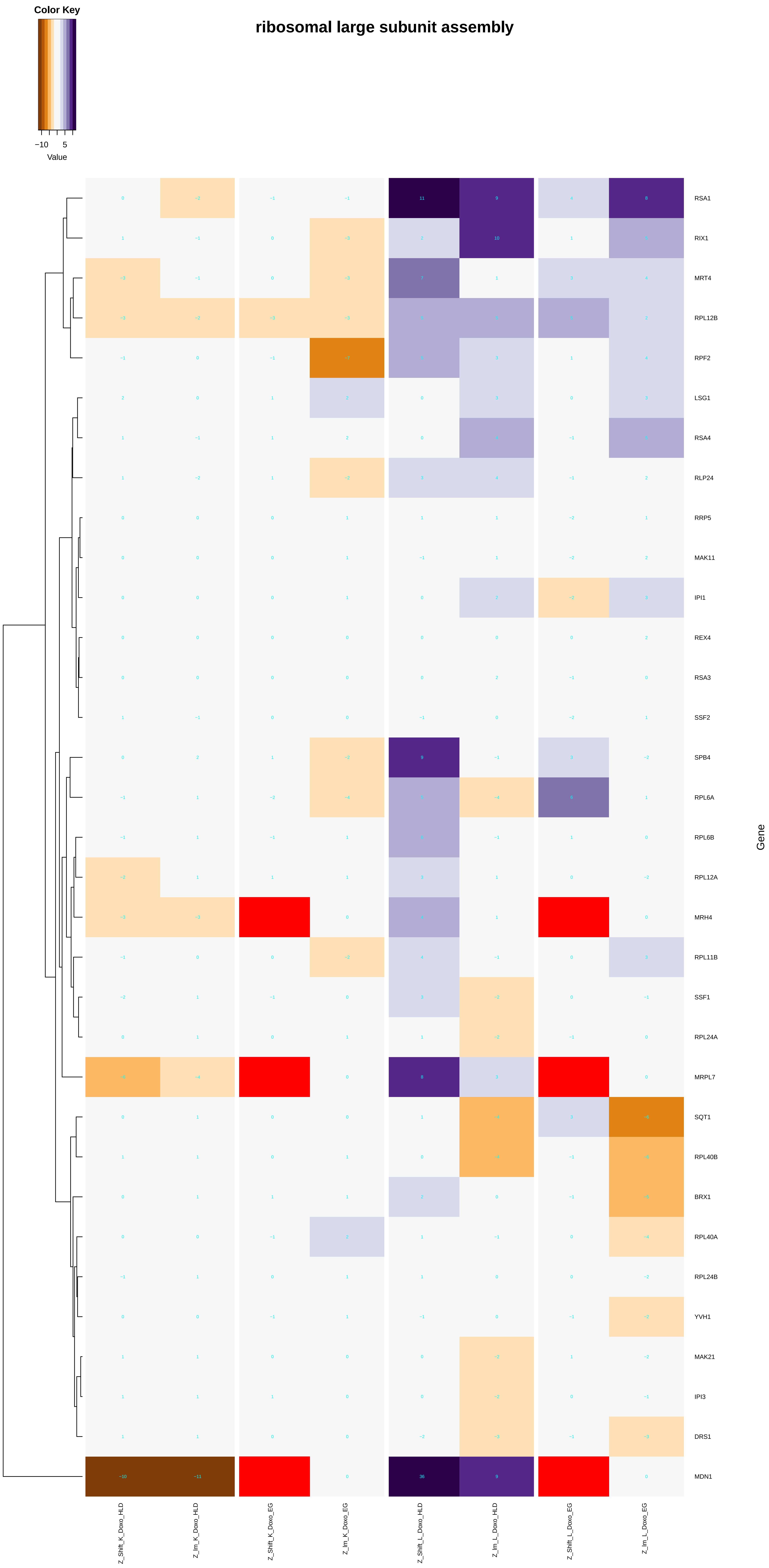

Color Key
−10
5
Value
ribosomal large subunit assembly
RSA1
RIX1
MRT4
RPL12B
RPF2
LSG1
RSA4
RLP24
RRP5
MAK11
IPI1
REX4
RSA3
SSF2
SPB4
RPL6A
RPL6B
RPL12A
MRH4
RPL11B
SSF1
RPL24A
MRPL7
SQT1
RPL40B
BRX1
RPL40A
RPL24B
YVH1
MAK21
IPI3
DRS1
MDN1
Gene
Z_Shift_K_Doxo_HLD
Z_lm_K_Doxo_HLD
Z_Shift_K_Doxo_EG
Z_lm_K_Doxo_EG
Z_Shift_L_Doxo_HLD
Z_lm_L_Doxo_HLD
Z_Shift_L_Doxo_EG
Z_lm_L_Doxo_EG

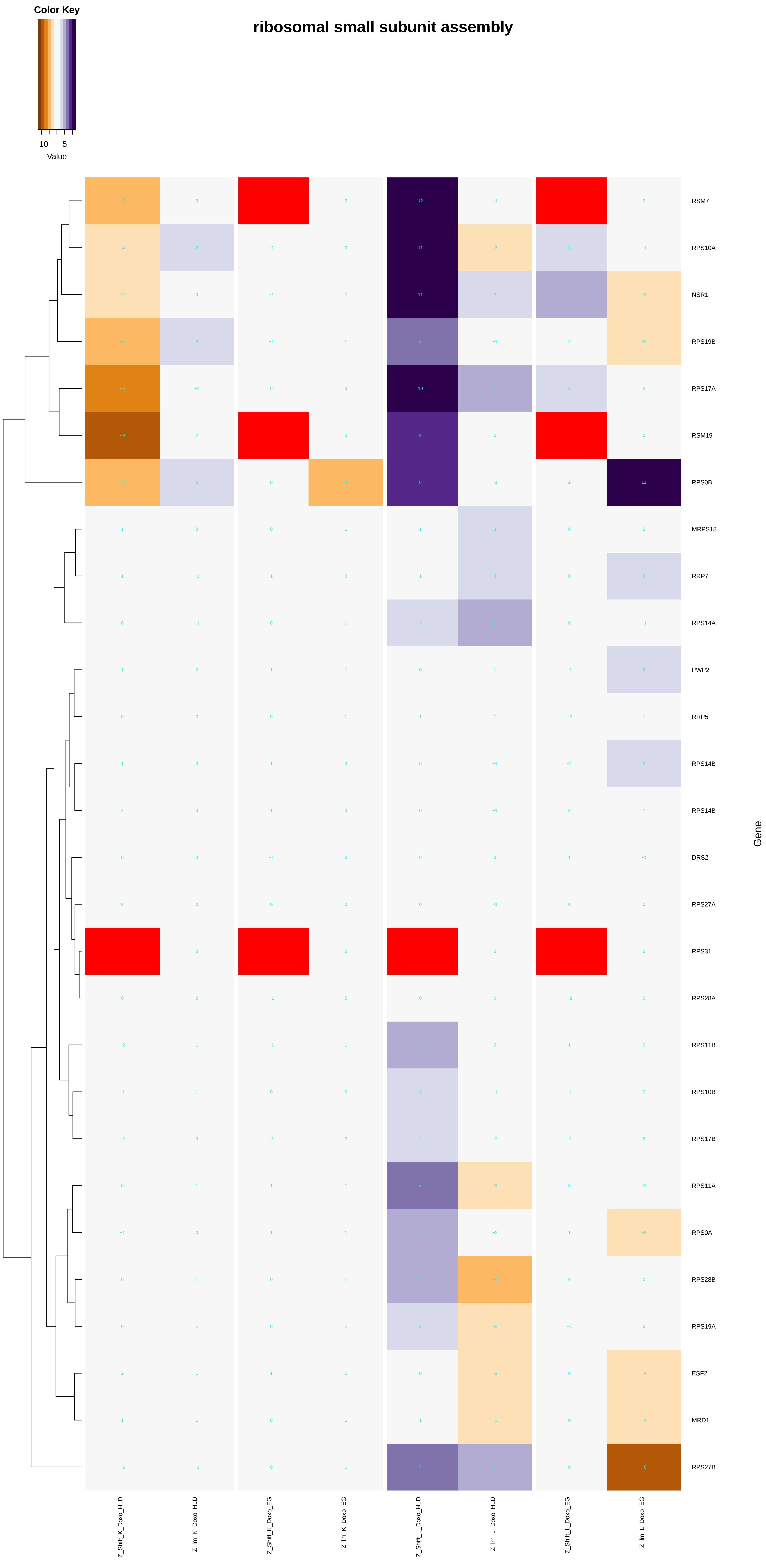

Color Key
−10
5
Value
ribosomal small subunit assembly
RSM7
RPS10A
NSR1
RPS19B
RPS17A
RSM19
RPS0B
MRPS18
RRP7
RPS14A
PWP2
RRP5
RPS14B
RPS14B
DRS2
RPS27A
RPS31
RPS28A
RPS11B
RPS10B
RPS17B
RPS11A
RPS0A
RPS28B
RPS19A
ESF2
MRD1
RPS27B
Gene
Z_Shift_K_Doxo_HLD
Z_Im_K_Doxo_HLD
Z_Shift_K_Doxo_EG
Z_Im_K_Doxo_EG
Z_Shift_L_Doxo_HLD
Z_Im_L_Doxo_HLD
Z_Shift_L_Doxo_EG
Z_Im_L_Doxo_EG

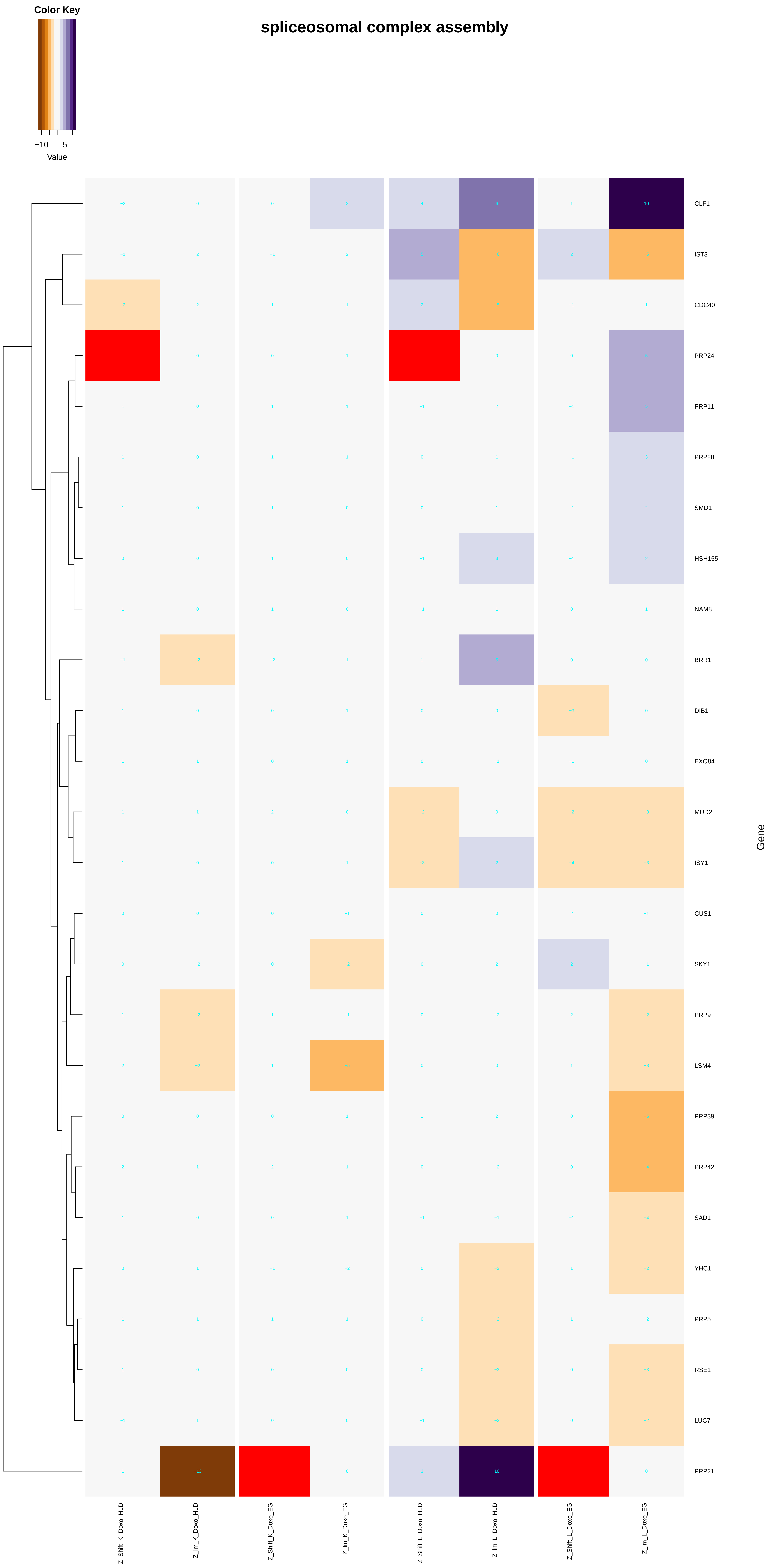
Color Key
−10
5
Value
spliceosomal complex assembly
CLF1
IST3
CDC40
PRP24
PRP11
PRP28
SMD1
HSH155
NAM8
BRR1
DIB1
EXO84
MUD2
ISY1
CUS1
SKY1
PRP9
LSM4
PRP39
PRP42
SAD1
YHC1
PRP5
RSE1
LUC7
PRP21
Gene
Z_Shift_K_Doxo_HLD
Z_Im_K_Doxo_HLD
Z_Shift_K_Doxo_EG
Z_Im_K_Doxo_EG
Z_Shift_L_Doxo_HLD
Z_Im_L_Doxo_HLD
Z_Shift_L_Doxo_EG
Z_Im_L_Doxo_EG

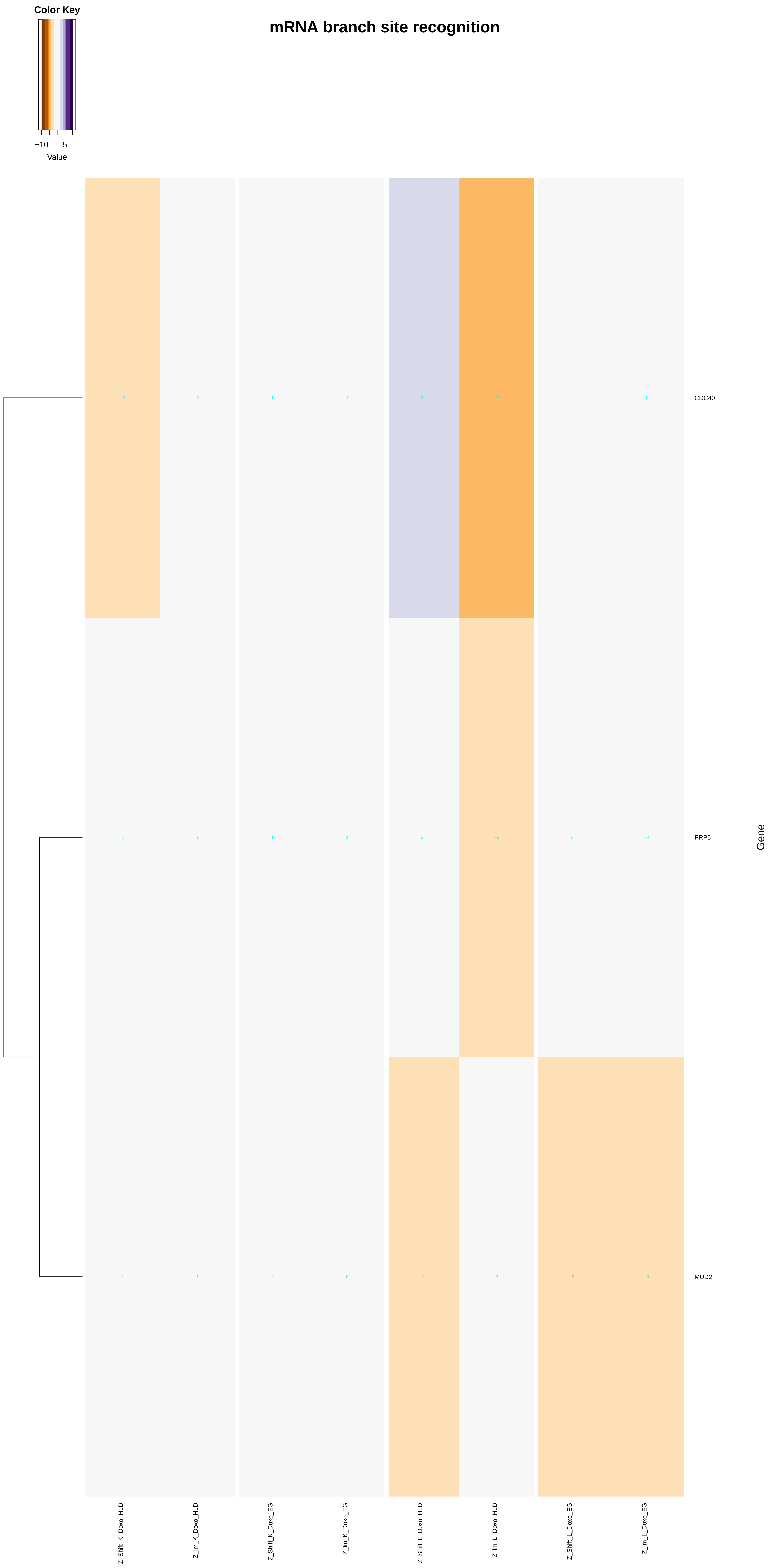
Color Key
−10
5
Value
mRNA branch site recognition
CDC40
PRP5
MUD2
Gene
Z_Shift_K_Doxo_HLD
Z_lm_K_Doxo_HLD
Z_Shift_K_Doxo_EG
Z_lm_K_Doxo_EG
Z_Shift_L_Doxo_HLD
Z_lm_L_Doxo_HLD
Z_Shift_L_Doxo_EG
Z_lm_L_Doxo_EG

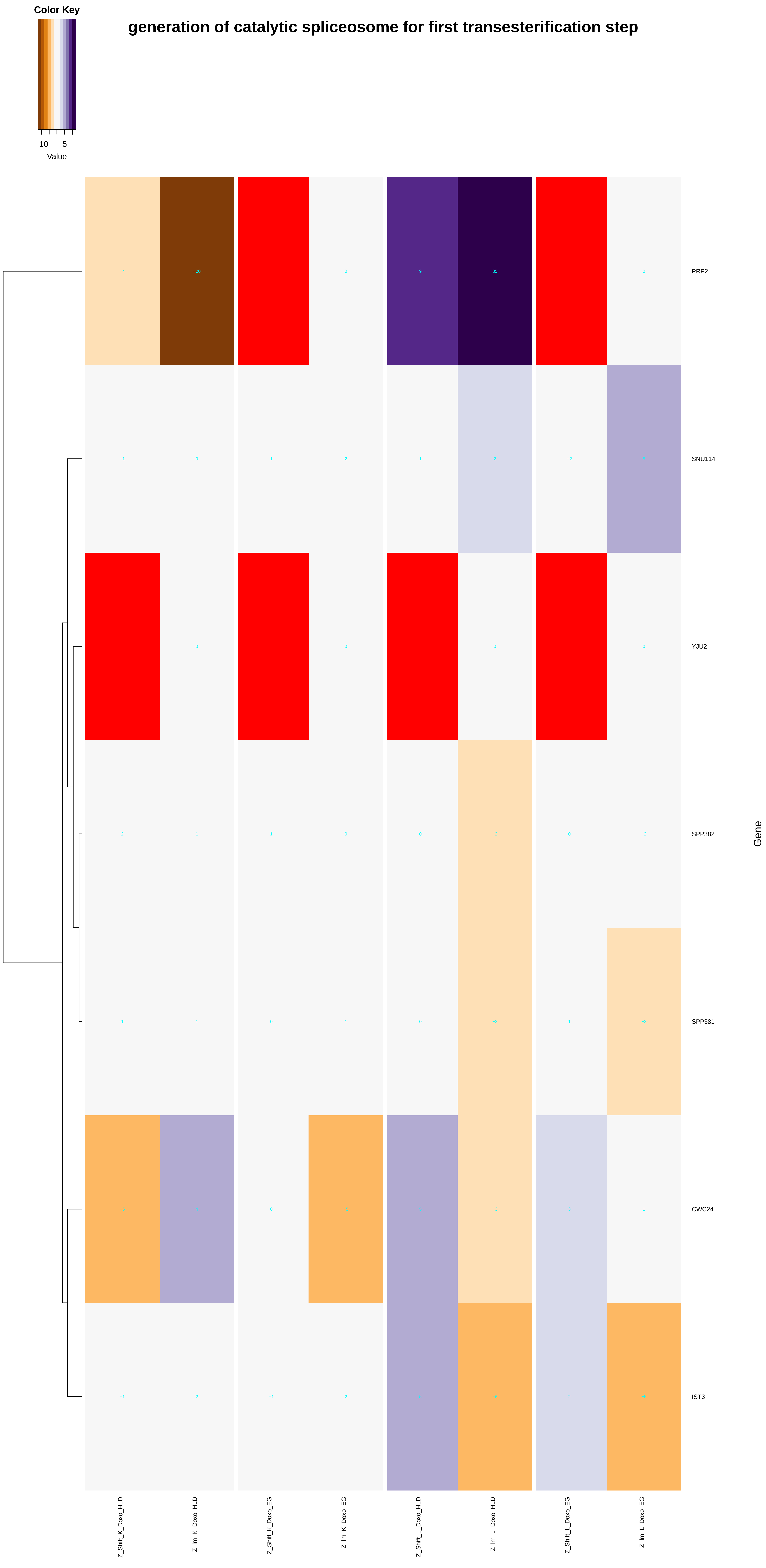

Color Key
−10 5
Value
generation of catalytic spliceosome for first transesterification step
PRP2
SNU114
YJU2
SPP382
SPP381
CWC24
IST3
Gene
Z_Shift_K_Doxo_HLD
Z_Im_K_Doxo_HLD
Z_Shift_K_Doxo_EG
Z_Im_K_Doxo_EG
Z_Shift_L_Doxo_HLD
Z_Im_L_Doxo_HLD
Z_Shift_L_Doxo_EG
Z_Im_L_Doxo_EG

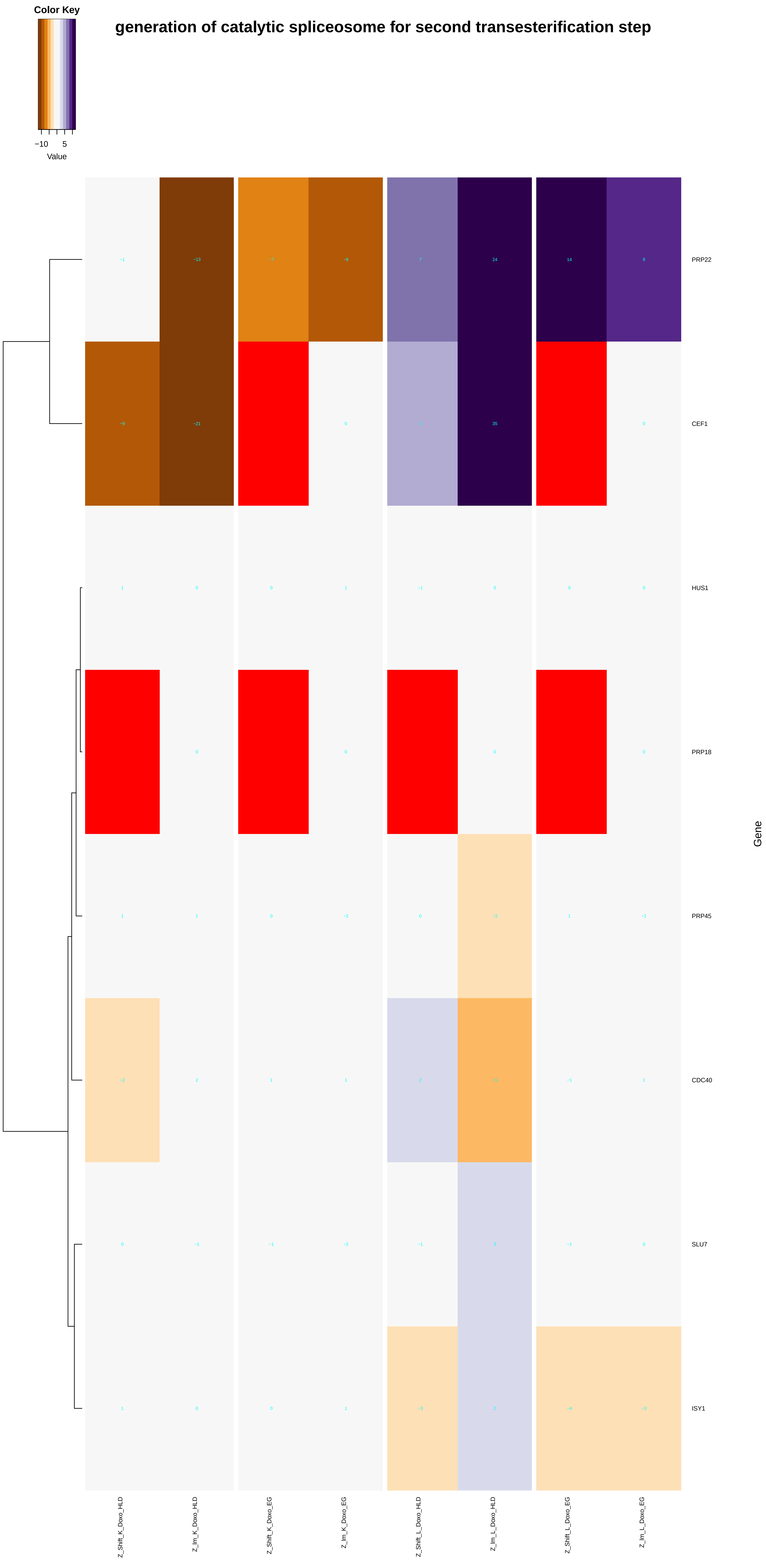

Color Key
−10
5
Value
generation of catalytic spliceosome for second transesterification step
PRP22
CEF1
HUS1
PRP18
PRP45
CDC40
SLU7
ISY1
Gene
Z_Shift_K_Doxo_HLD
Z_Im_K_Doxo_HLD
Z_Shift_K_Doxo_EG
Z_Im_K_Doxo_EG
Z_Shift_L_Doxo_HLD
Z_Im_L_Doxo_HLD
Z_Shift_L_Doxo_EG
Z_Im_L_Doxo_EG

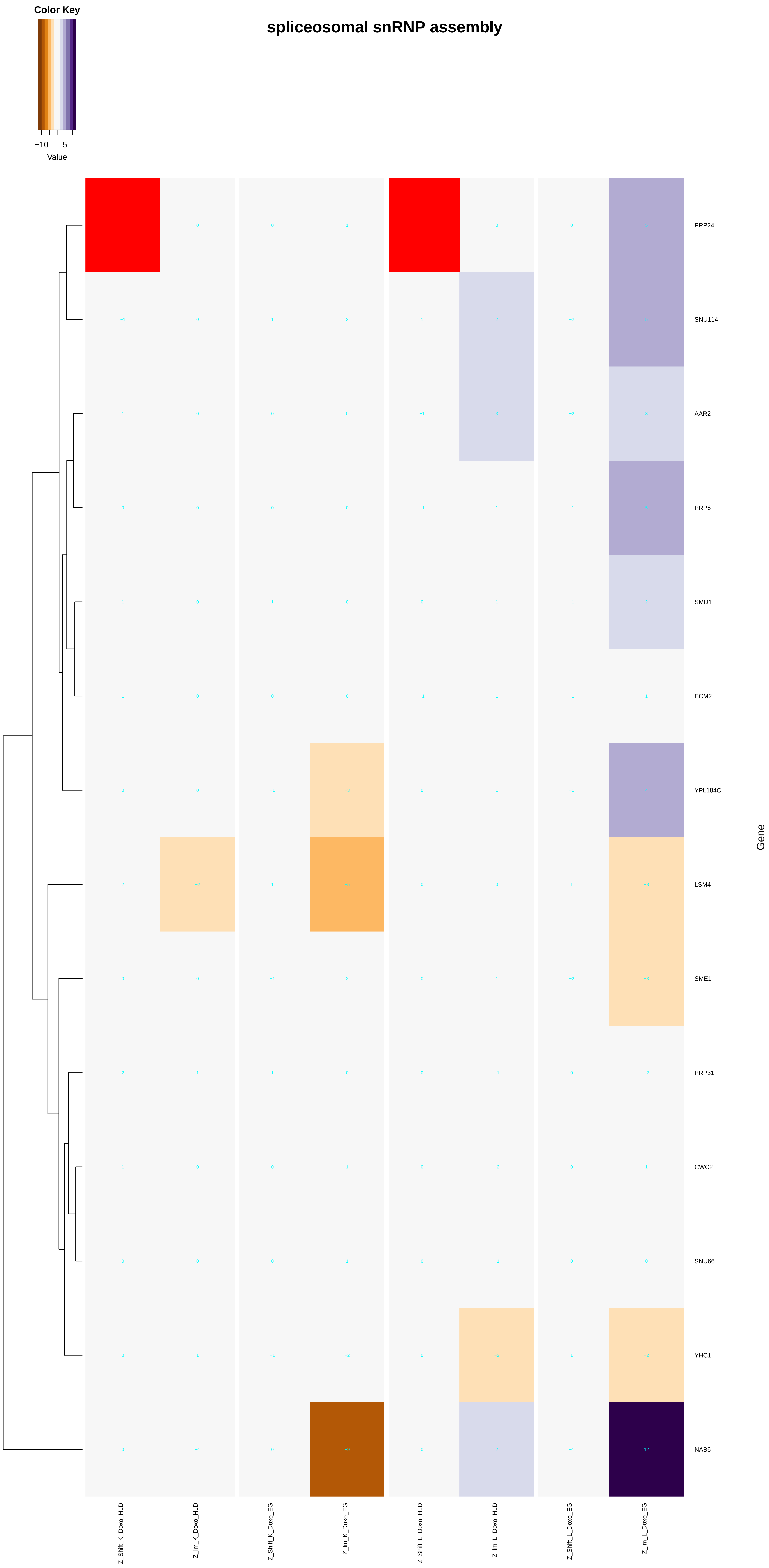
Color Key
spliceosomal snRNP assembly
−10
5
Value
PRP24
SNU114
AAR2
PRP6
SMD1
ECM2
YPL184C
LSM4
SME1
PRP31
CWC2
SNU66
YHC1
NAB6
Gene
Z_Shift_K_Doxo_HLD
Z_lm_K_Doxo_HLD
Z_Shift_K_Doxo_EG
Z_lm_K_Doxo_EG
Z_Shift_L_Doxo_HLD
Z_lm_L_Doxo_HLD
Z_Shift_L_Doxo_EG
Z_lm_L_Doxo_EG

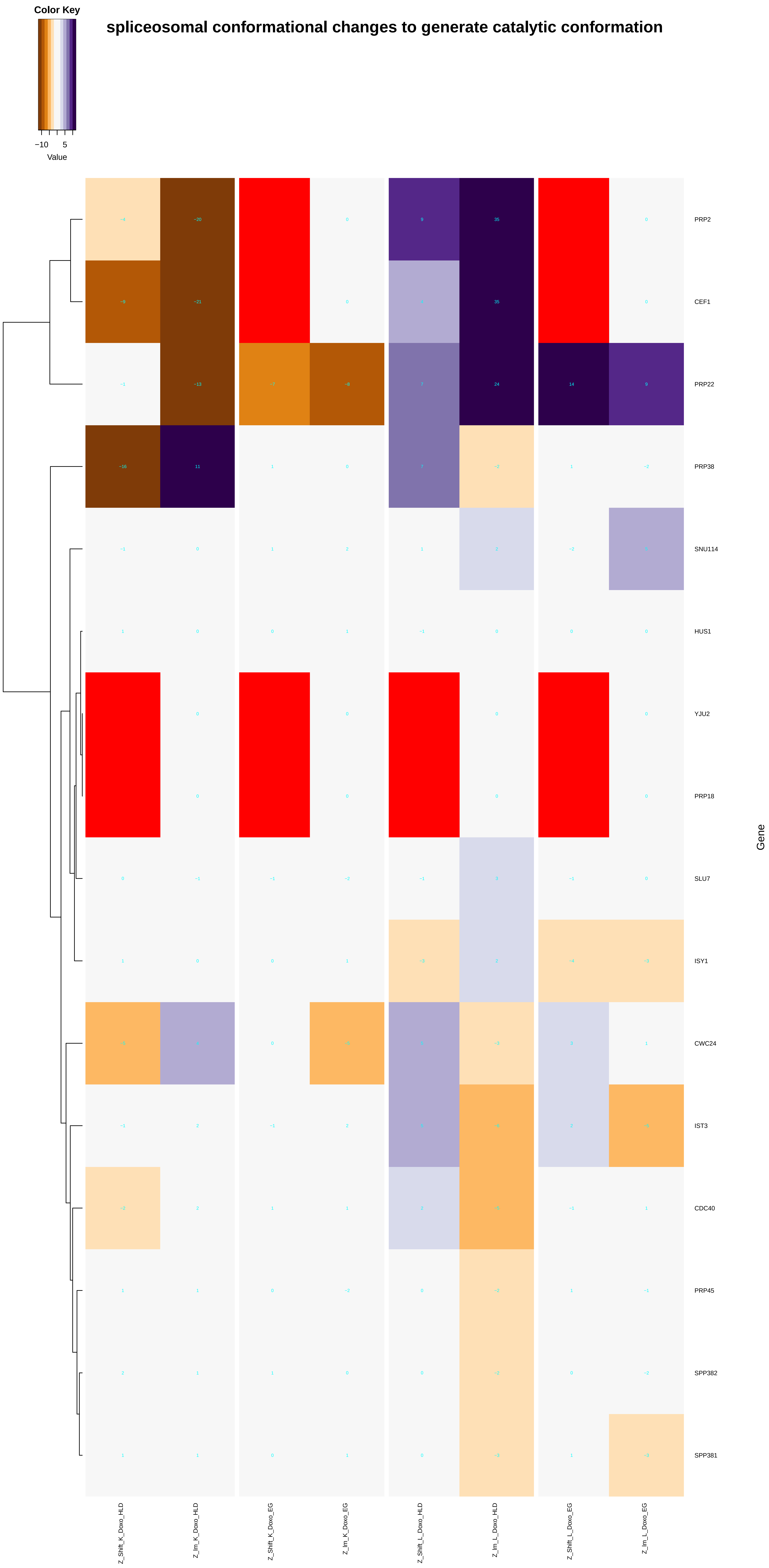

Color Key
−10
5
Value
spliceosomal conformational changes to generate catalytic conformation
PRP2
CEF1
PRP22
PRP38
SNU114
HUS1
YJU2
PRP18
SLU7
ISY1
CWC24
IST3
CDC40
PRP45
SPP382
SPP381
Gene
Z_Shift_K_Doxo_HLD
Z_Im_K_Doxo_HLD
Z_Shift_K_Doxo_EG
Z_Im_K_Doxo_EG
Z_Shift_L_Doxo_HLD
Z_Im_L_Doxo_HLD
Z_Shift_L_Doxo_EG
Z_Im_L_Doxo_EG

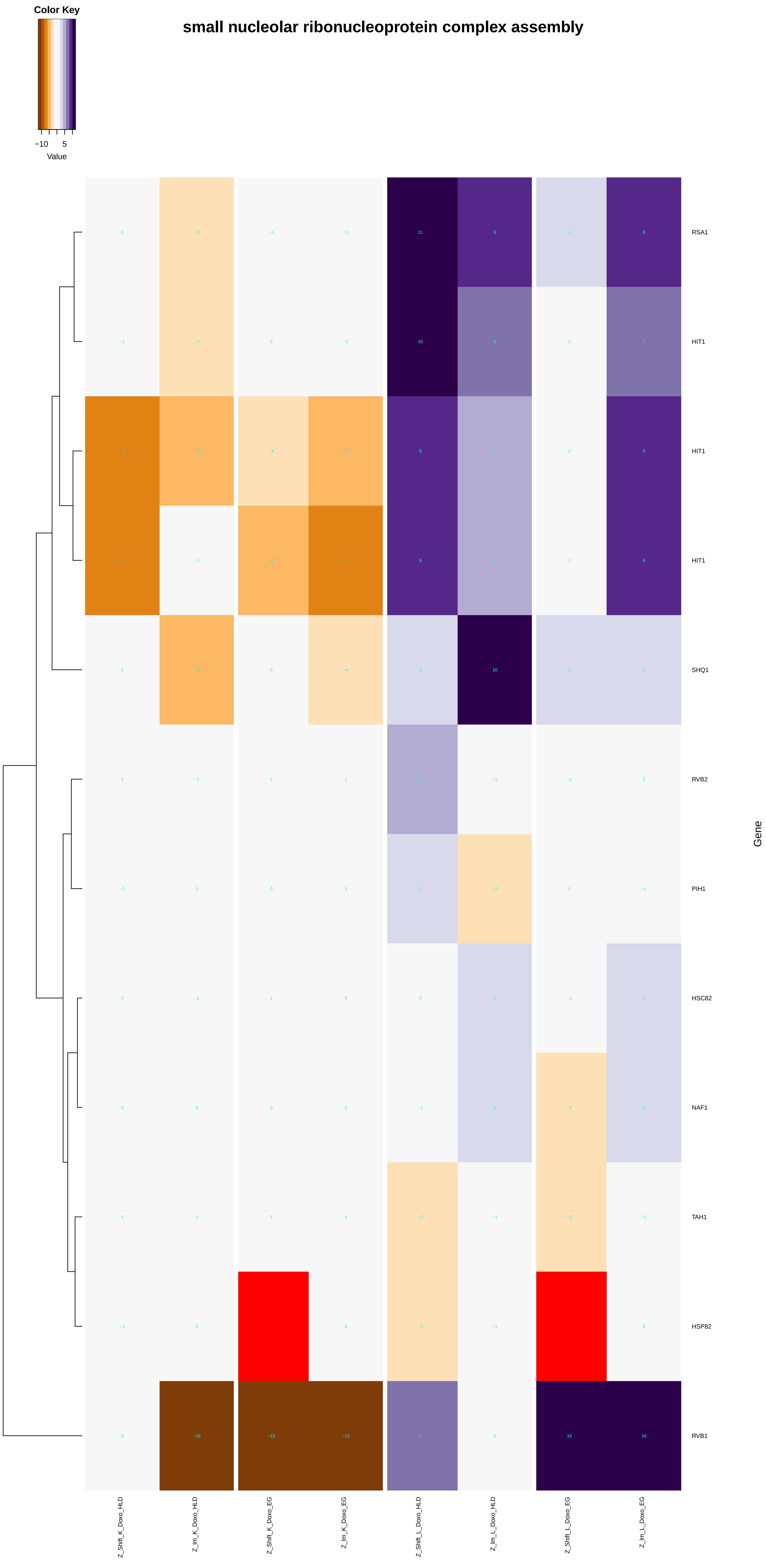
Color Key
−10
5
Value
small nucleolar ribonucleoprotein complex assembly
RSA1
HIT1
HIT1
HIT1
SHQ1
RVB2
PIH1
HSC82
NAF1
TAH1
HSP82
RVB1
Gene
Z_Shift_K_Doxo_HLD
Z_Im_K_Doxo_HLD
Z_Shift_K_Doxo_EG
Z_Im_K_Doxo_EG
Z_Shift_L_Doxo_HLD
Z_Im_L_Doxo_HLD
Z_Shift_L_Doxo_EG
Z_Im_L_Doxo_EG

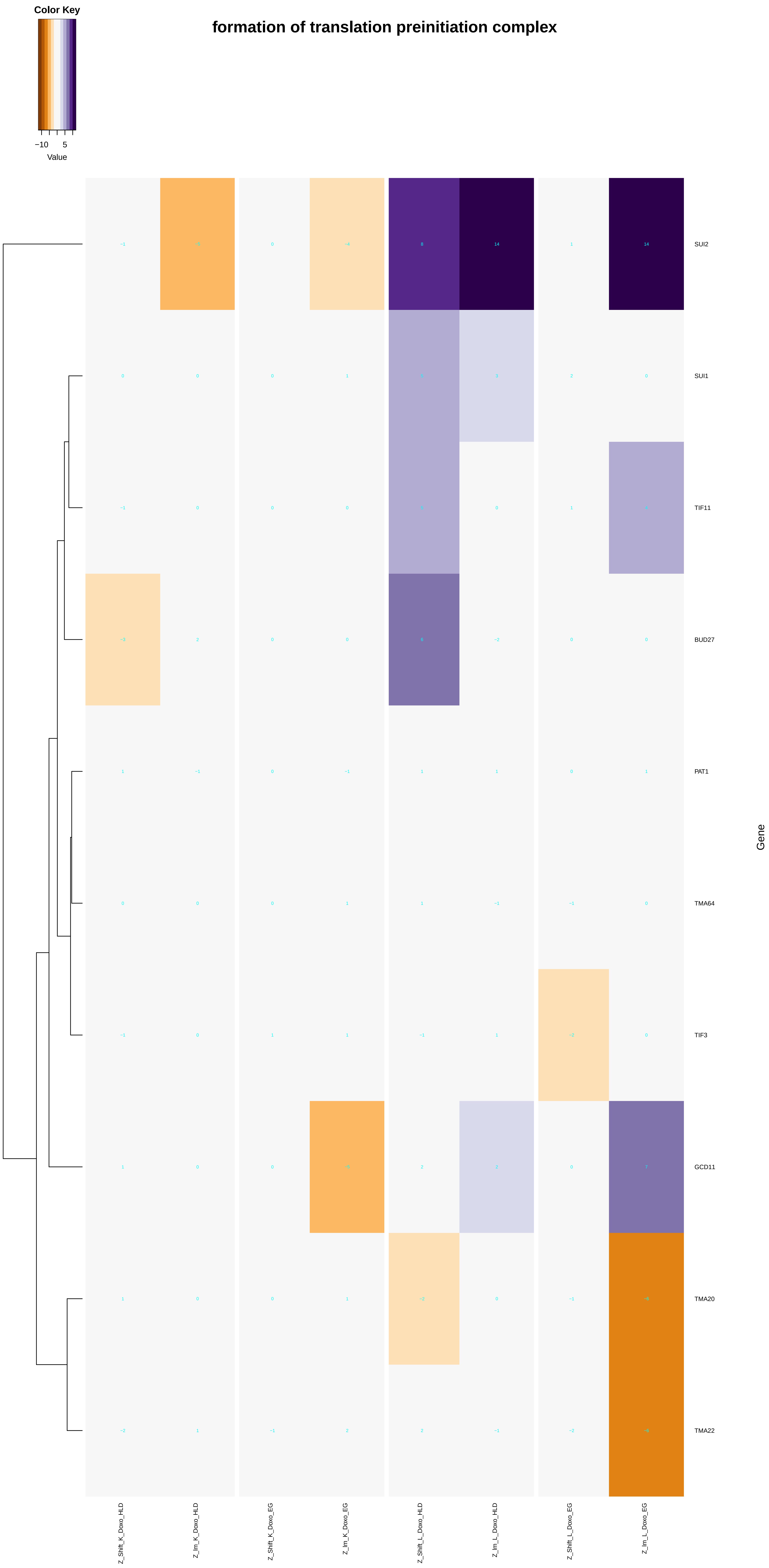
Color Key
−10
5
Value
formation of translation preinitiation complex
SUI2
SUI1
TIF11
BUD27
PAT1
TMA64
TIF3
GCD11
TMA20
TMA22
Gene
Z_Shift_K_Doxo_HLD
Z_lm_K_Doxo_HLD
Z_Shift_K_Doxo_EG
Z_lm_K_Doxo_EG
Z_Shift_L_Doxo_HLD
Z_lm_L_Doxo_HLD
Z_Shift_L_Doxo_EG
Z_lm_L_Doxo_EG

formation of cytoplasmic translation initiation complex

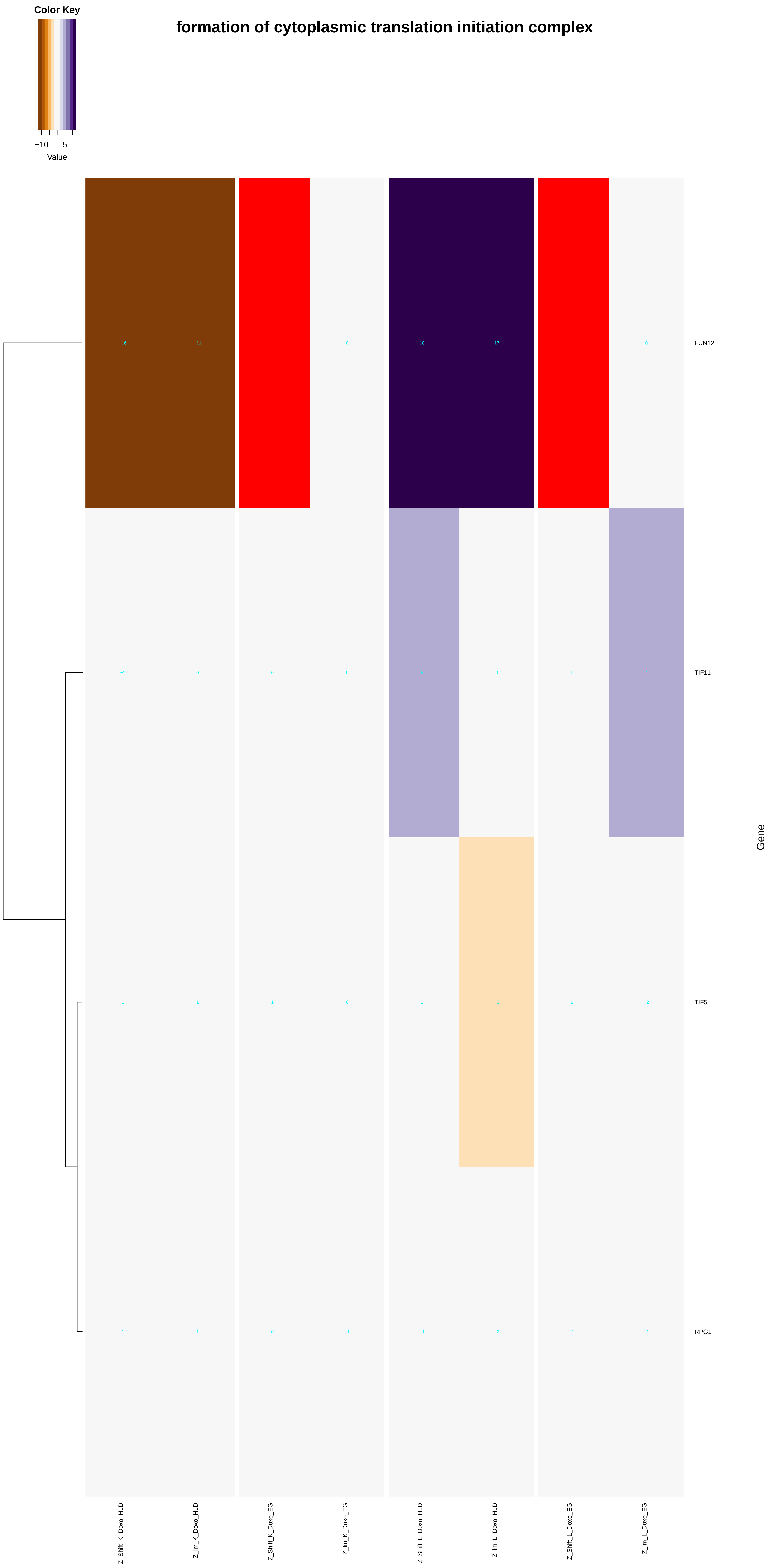

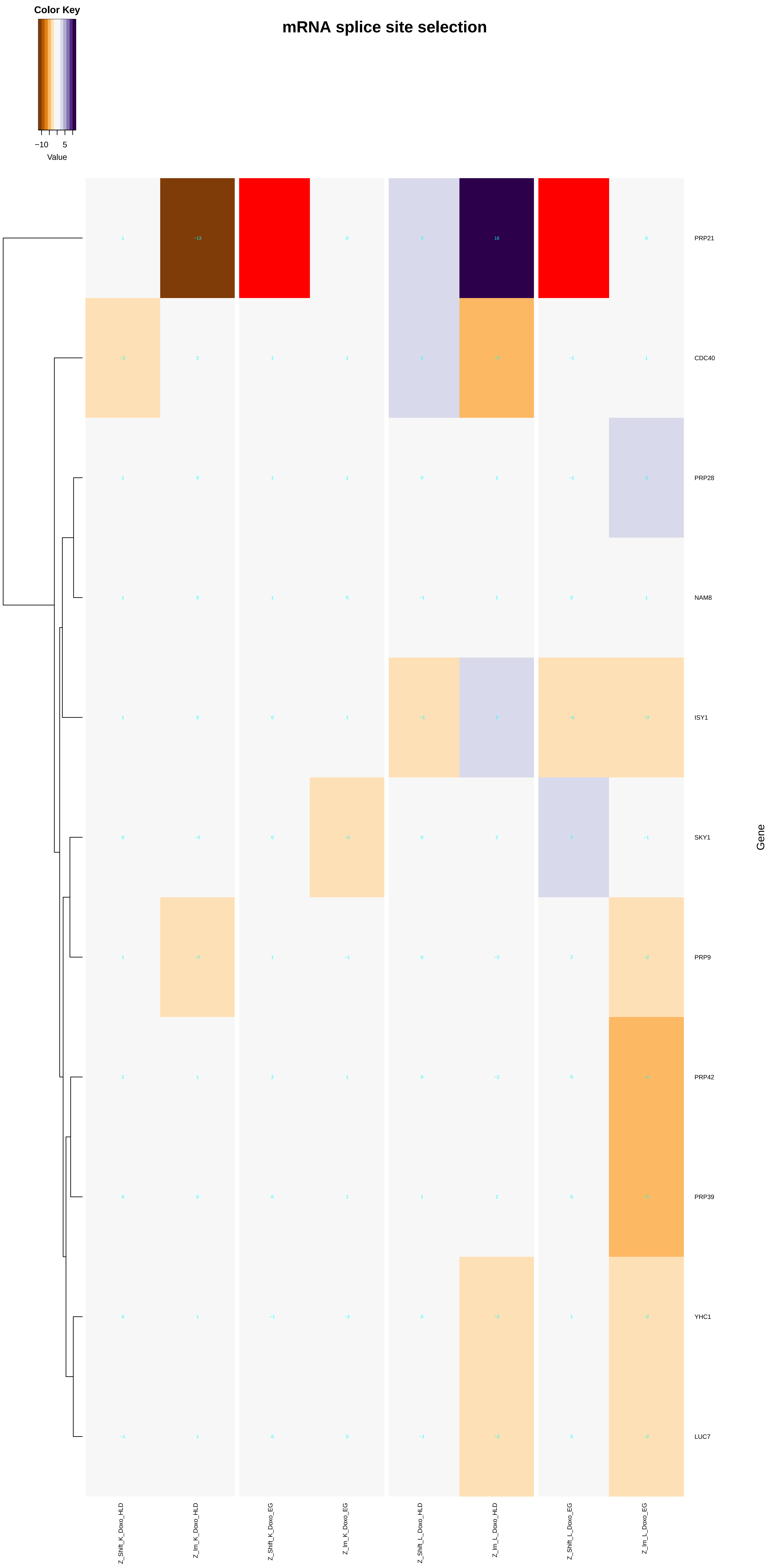
Color Key
−10
5
Value
mRNA splice site selection
PRP21
CDC40
PRP28
NAM8
ISY1
SKY1
PRP9
PRP42
PRP39
YHC1
LUC7
Gene
Z_Shift_K_Doxo_HLD
Z_lm_K_Doxo_HLD
Z_Shift_K_Doxo_EG
Z_lm_K_Doxo_EG
Z_Shift_L_Doxo_HLD
Z_lm_L_Doxo_HLD
Z_Shift_L_Doxo_EG
Z_lm_L_Doxo_EG

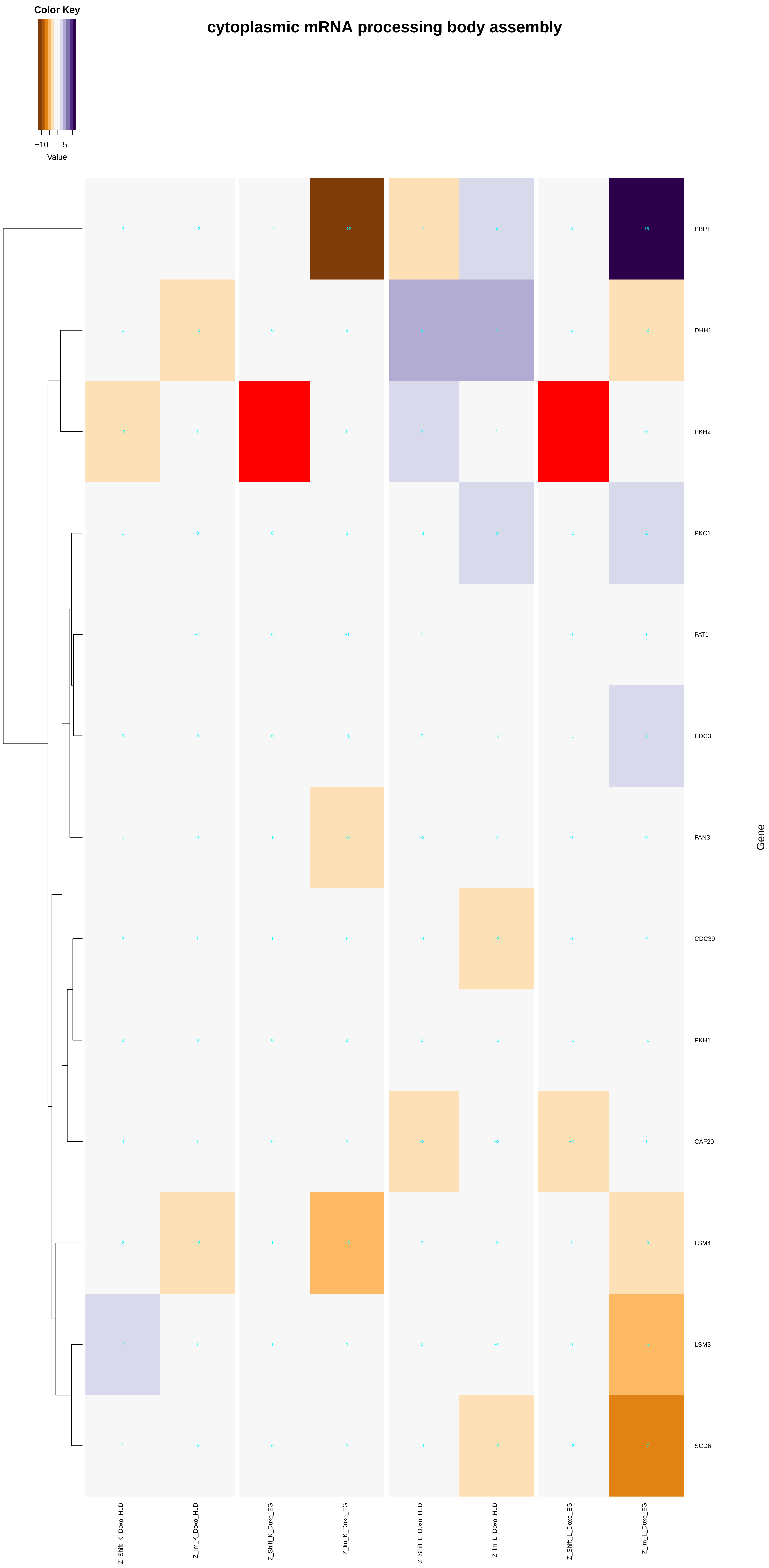
Color Key
−10
5
Value
cytoplasmic mRNA processing body assembly
PBP1
DHH1
PKH2
PKC1
PAT1
EDC3
PAN3
CDC39
PKH1
CAF20
LSM4
LSM3
SCD6
Gene
Z_Shift_K_Doxo_HLD
Z_Im_K_Doxo_HLD
Z_Shift_K_Doxo_EG
Z_Im_K_Doxo_EG
Z_Shift_L_Doxo_HLD
Z_Im_L_Doxo_HLD
Z_Shift_L_Doxo_EG
Z_Im_L_Doxo_EG

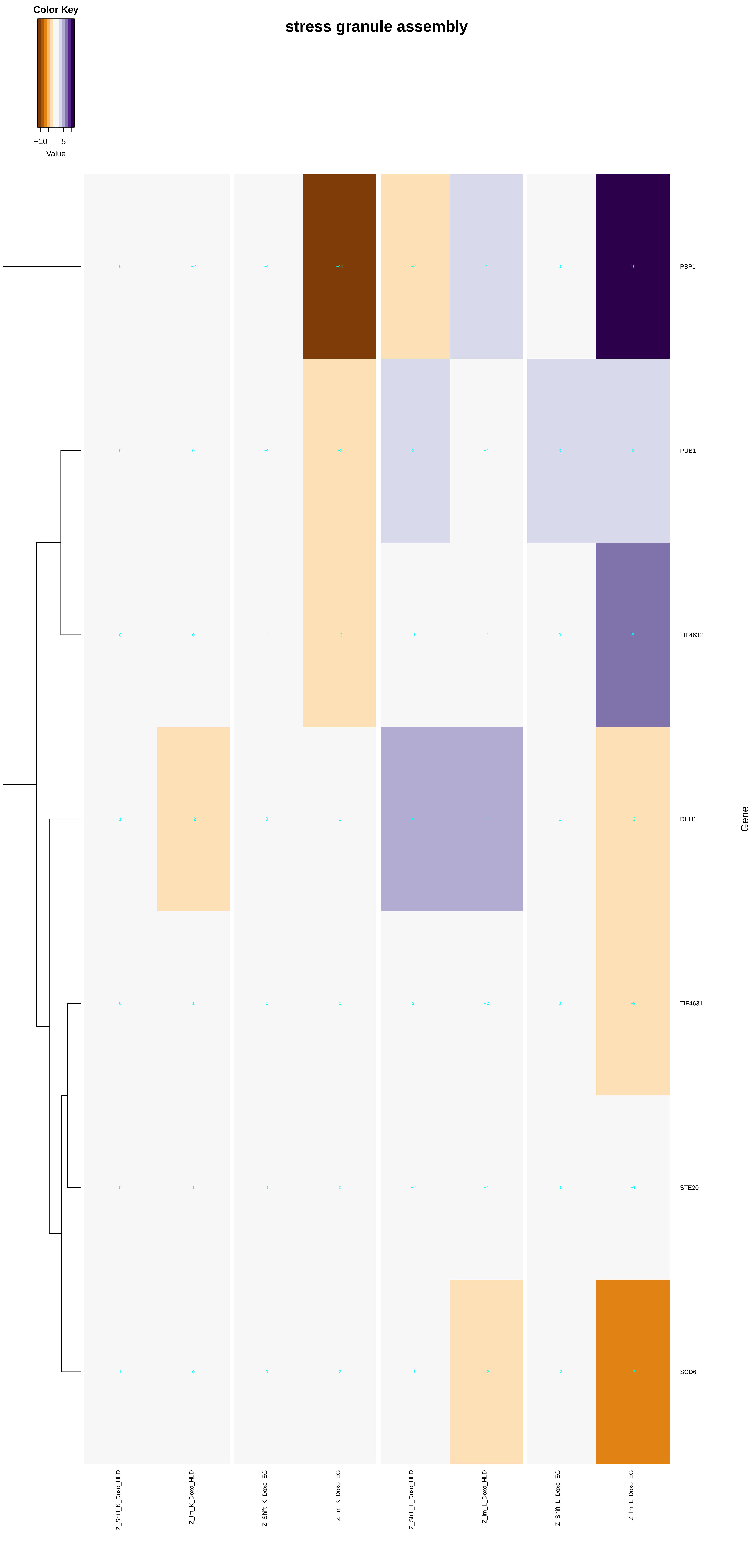
Color Key
−10
5
Value
stress granule assembly
PBP1
PUB1
TIF4632
DHH1
TIF4631
STE20
SCD6
Gene
Z_Shift_K_Doxo_HLD
Z_Im_K_Doxo_HLD
Z_Shift_K_Doxo_EG
Z_Im_K_Doxo_EG
Z_Shift_L_Doxo_HLD
Z_Im_L_Doxo_HLD
Z_Shift_L_Doxo_EG
Z_Im_L_Doxo_EG

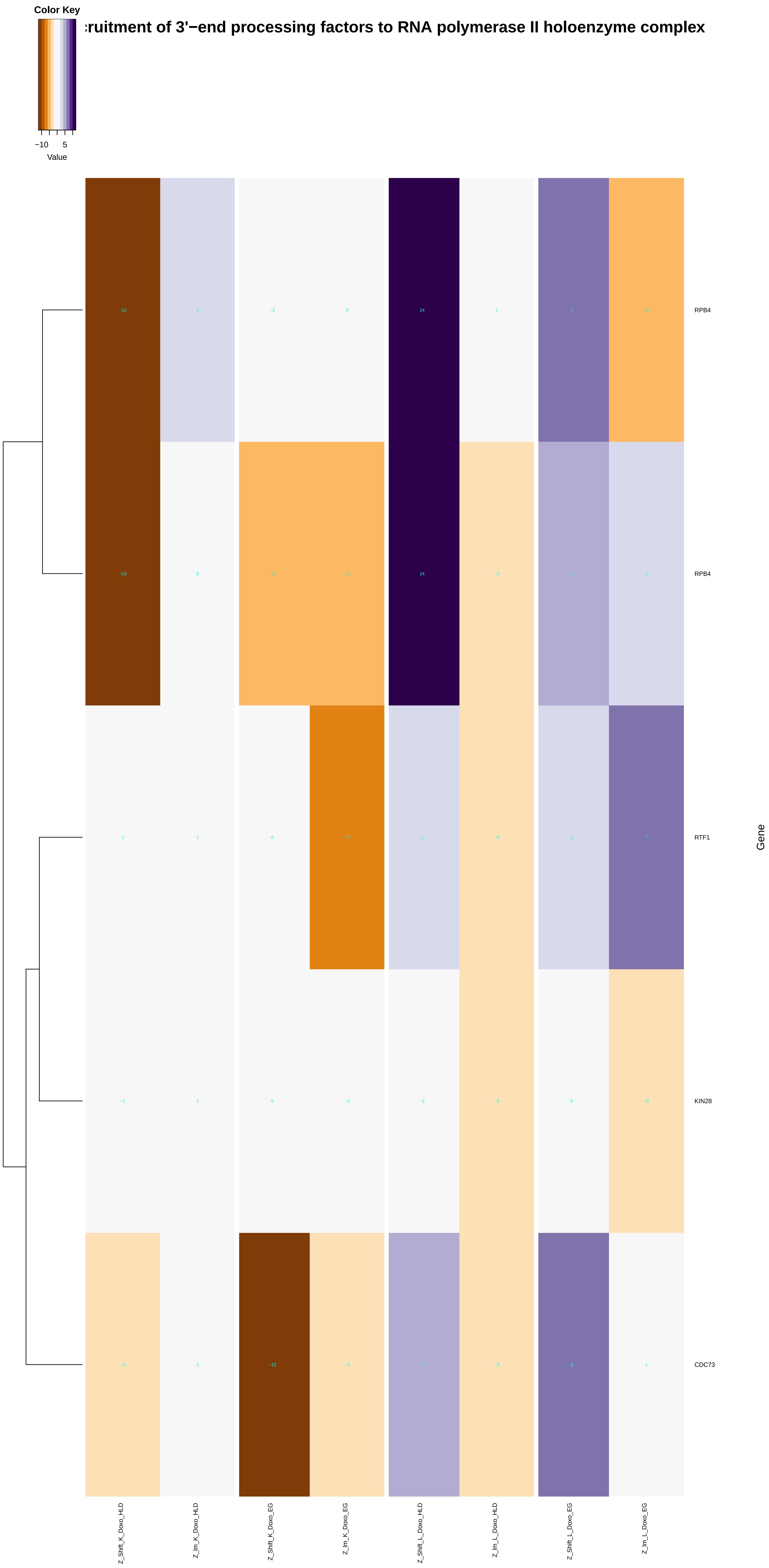

Color Key
−10
5
Value
:ruitment of 3'−end processing factors to RNA polymerase II holoenzyme complex
RPB4
RPB4
RTF1
KIN28
CDC73
Gene
Z_Shift_K_Doxo_HLD
Z_Im_K_Doxo_HLD
Z_Shift_K_Doxo_EG
Z_Im_K_Doxo_EG
Z_Shift_L_Doxo_HLD
Z_Im_L_Doxo_HLD
Z_Shift_L_Doxo_EG
Z_Im_L_Doxo_EG

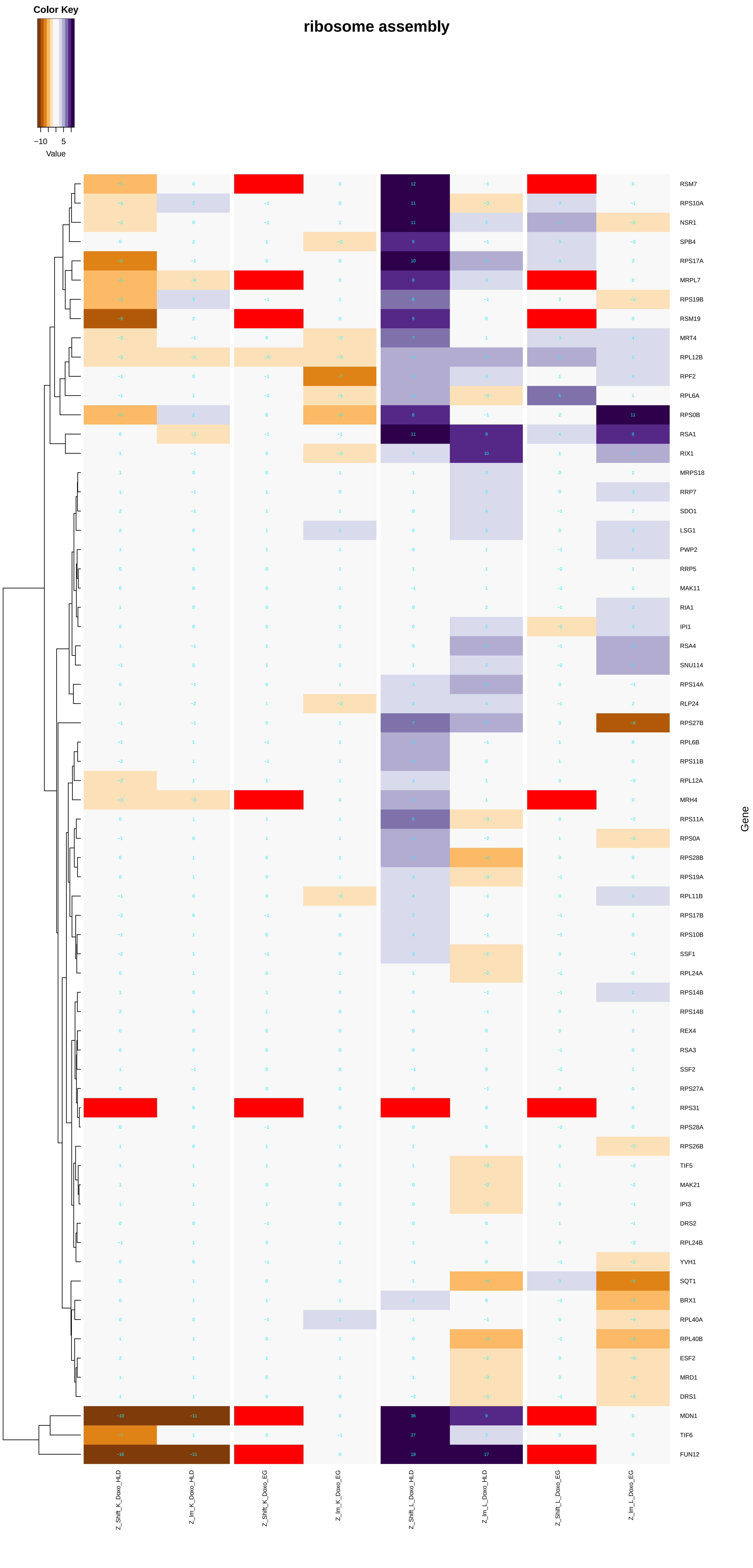

Color Key
−10
5
Value
ribosome assembly
RSM7
RPS10A
NSR1
SPB4
RPS17A
MRPL7
RPS19B
RSM19
MRT4
RPL12B
RPF2
RPL6A
RPS0B
RSA1
RIX1
MRPS18
RRP7
SDO1
LSG1
PWP2
RRP5
MAK11
RIA1
IPI1
RSA4
SNU114
RPS14A
RLP24
RPS27B
RPL6B
RPS11B
RPL12A
MRH4
RPS11A
RPS0A
RPS28B
RPS19A
RPL11B
RPS17B
RPS10B
SSF1
RPL24A
RPS14B
RPS14B
REX4
RSA3
SSF2
RPS27A
RPS31
RPS28A
RPS26B
TIF5
MAK21
IPI3
DRS2
RPL24B
YVH1
SQT1
BRX1
RPL40A
RPL40B
ESF2
MRD1
DRS1
MDN1
TIF6
FUN12
Gene
Z_Shift_K_Doxo_HLD
Z_Im_K_Doxo_HLD
Z_Shift_K_Doxo_EG
Z_Im_K_Doxo_EG
Z_Shift_L_Doxo_HLD
Z_Im_L_Doxo_HLD
Z_Shift_L_Doxo_EG
Z_Im_L_Doxo_EG

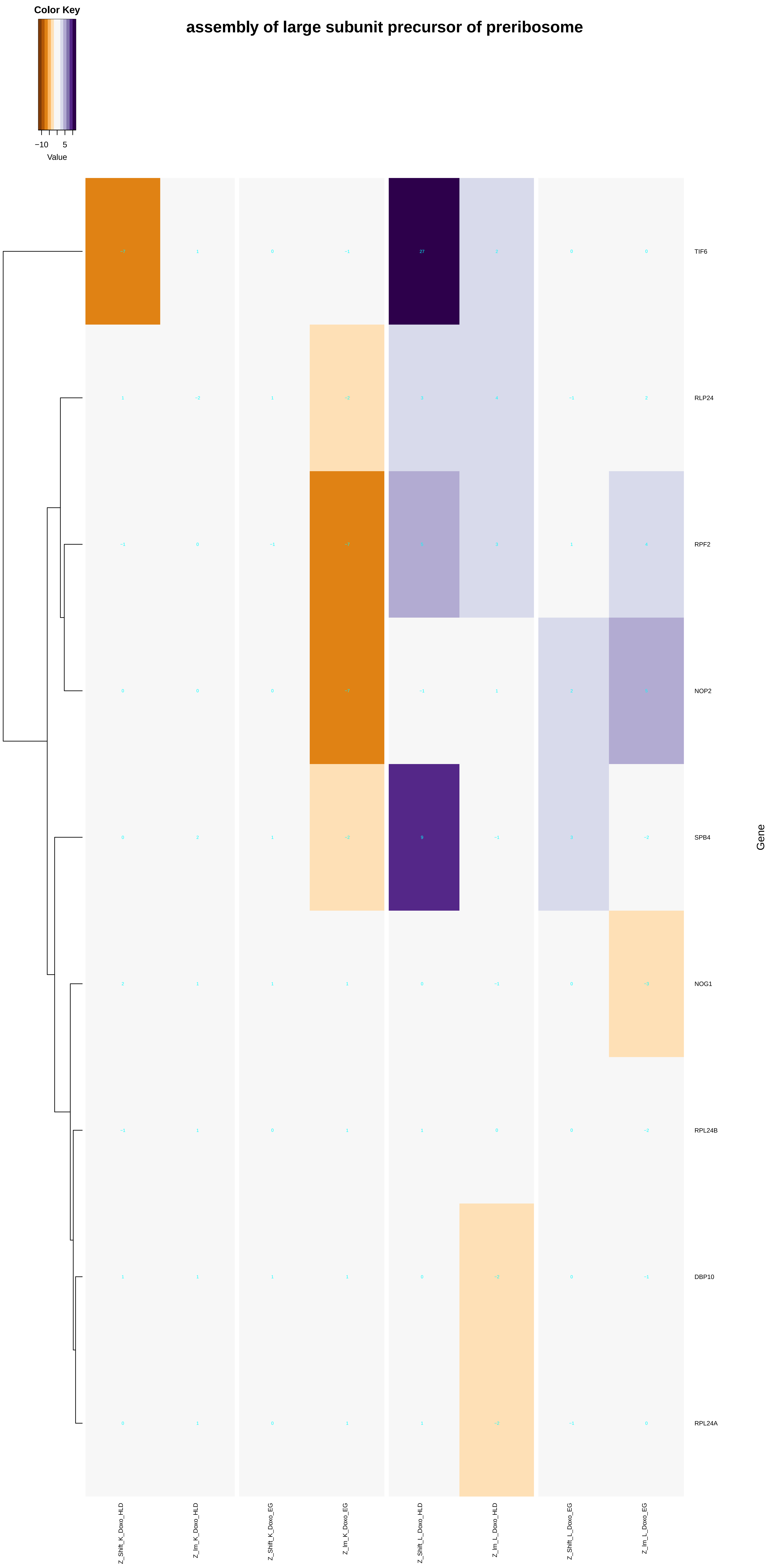
assembly of large subunit precursor of preribosome
Color Key
−10
5
Value
TIF6
RLP24
RPF2
NOP2
SPB4
NOG1
RPL24B
DBP10
RPL24A
Gene
Z_Shift_K_Doxo_HLD
Z_Im_K_Doxo_HLD
Z_Shift_K_Doxo_EG
Z_Im_K_Doxo_EG
Z_Shift_L_Doxo_HLD
Z_Im_L_Doxo_HLD
Z_Shift_L_Doxo_EG
Z_Im_L_Doxo_EG

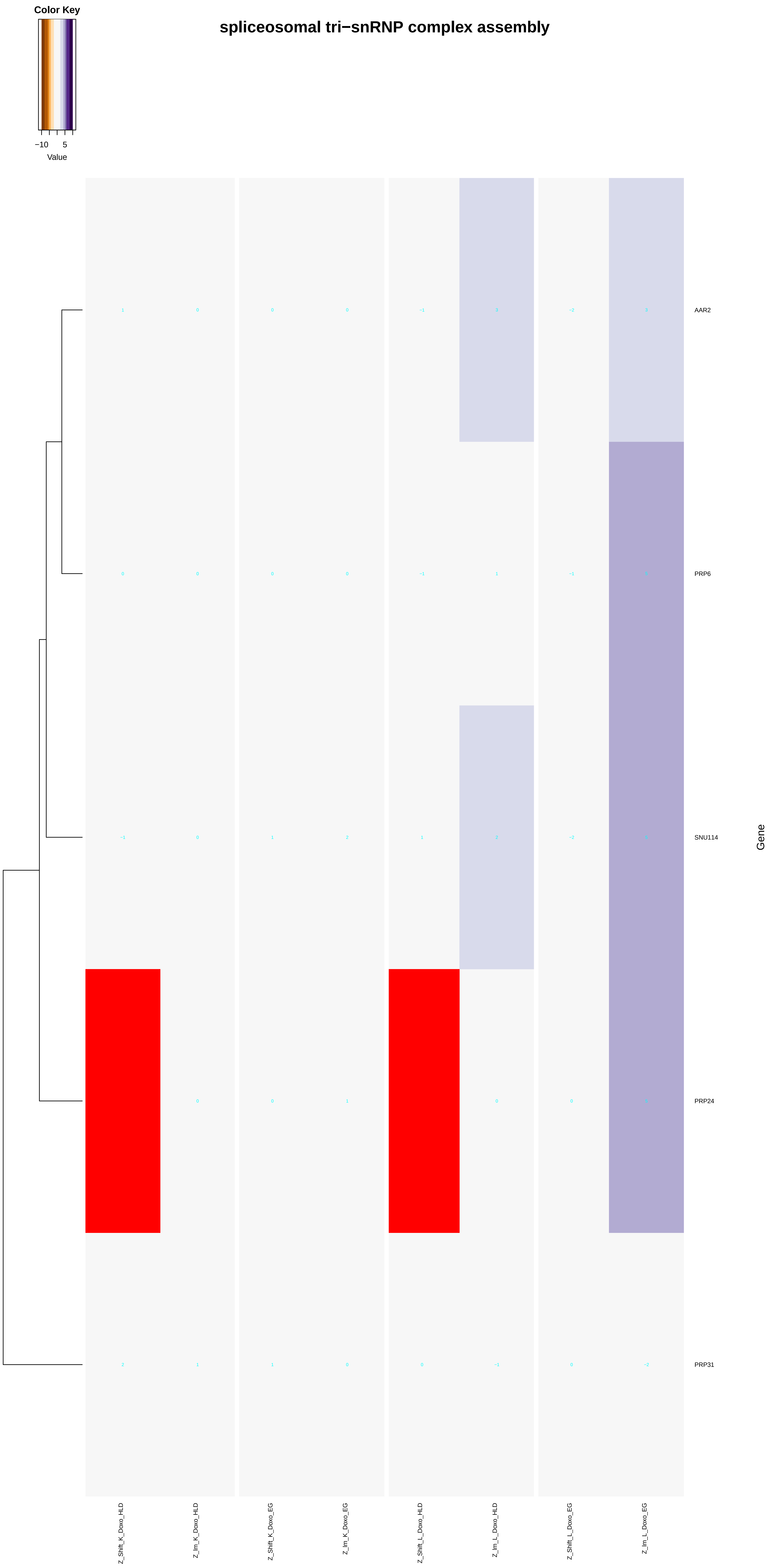
Color Key
−10
5
Value
spliceosomal tri−snRNP complex assembly
AAR2
PRP6
SNU114
PRP24
PRP31
Gene
Z_Shift_K_Doxo_HLD
Z_lm_K_Doxo_HLD
Z_Shift_K_Doxo_EG
Z_lm_K_Doxo_EG
Z_Shift_L_Doxo_HLD
Z_lm_L_Doxo_HLD
Z_Shift_L_Doxo_EG
Z_lm_L_Doxo_EG

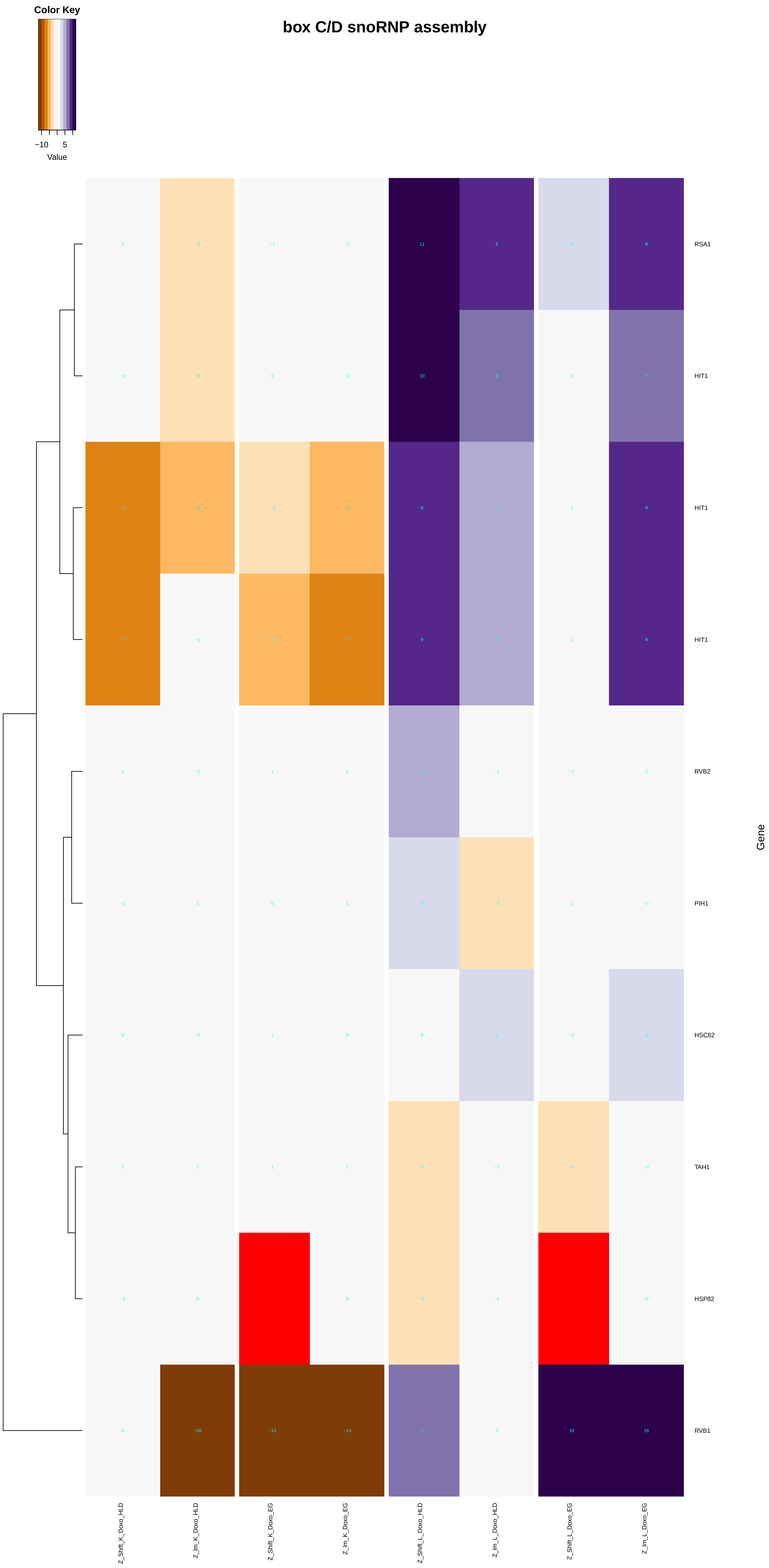
box C/D snoRNP assembly
Color Key
−10
5
Value
RSA1
HIT1
HIT1
HIT1
RVB2
PIH1
HSC82
TAH1
HSP82
RVB1
Gene
Z_Shift_K_Doxo_HLD
Z_lm_K_Doxo_HLD
Z_Shift_K_Doxo_EG
Z_lm_K_Doxo_EG
Z_Shift_L_Doxo_HLD
Z_lm_L_Doxo_HLD
Z_Shift_L_Doxo_EG
Z_lm_L_Doxo_EG

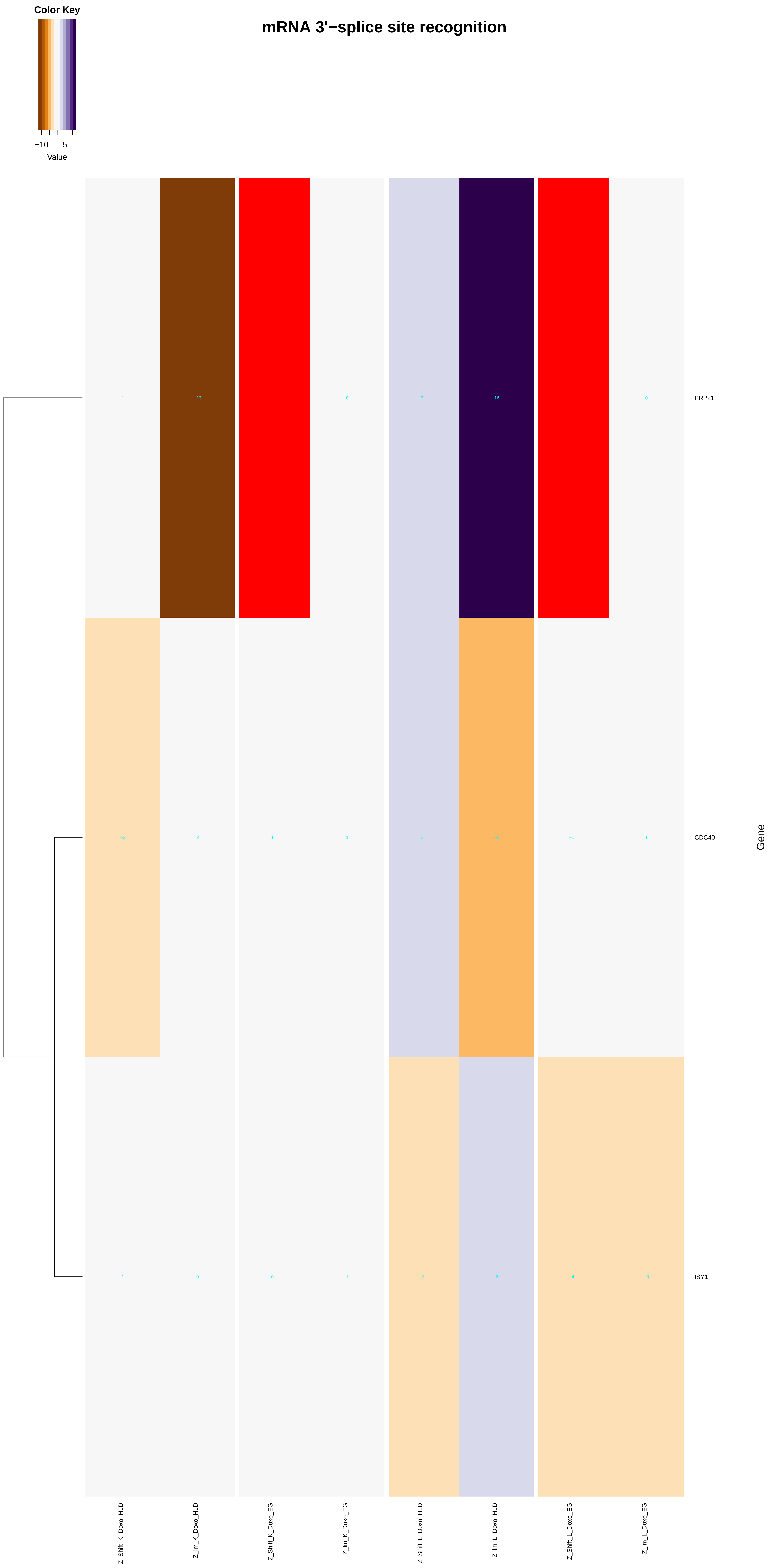
Color Key
−10
5
Value
mRNA 3'−splice site recognition
PRP21
CDC40
ISY1
Gene
Z_Shift_K_Doxo_HLD
Z_Im_K_Doxo_HLD
Z_Shift_K_Doxo_EG
Z_Im_K_Doxo_EG
Z_Shift_L_Doxo_HLD
Z_Im_L_Doxo_HLD
Z_Shift_L_Doxo_EG
Z_Im_L_Doxo_EG

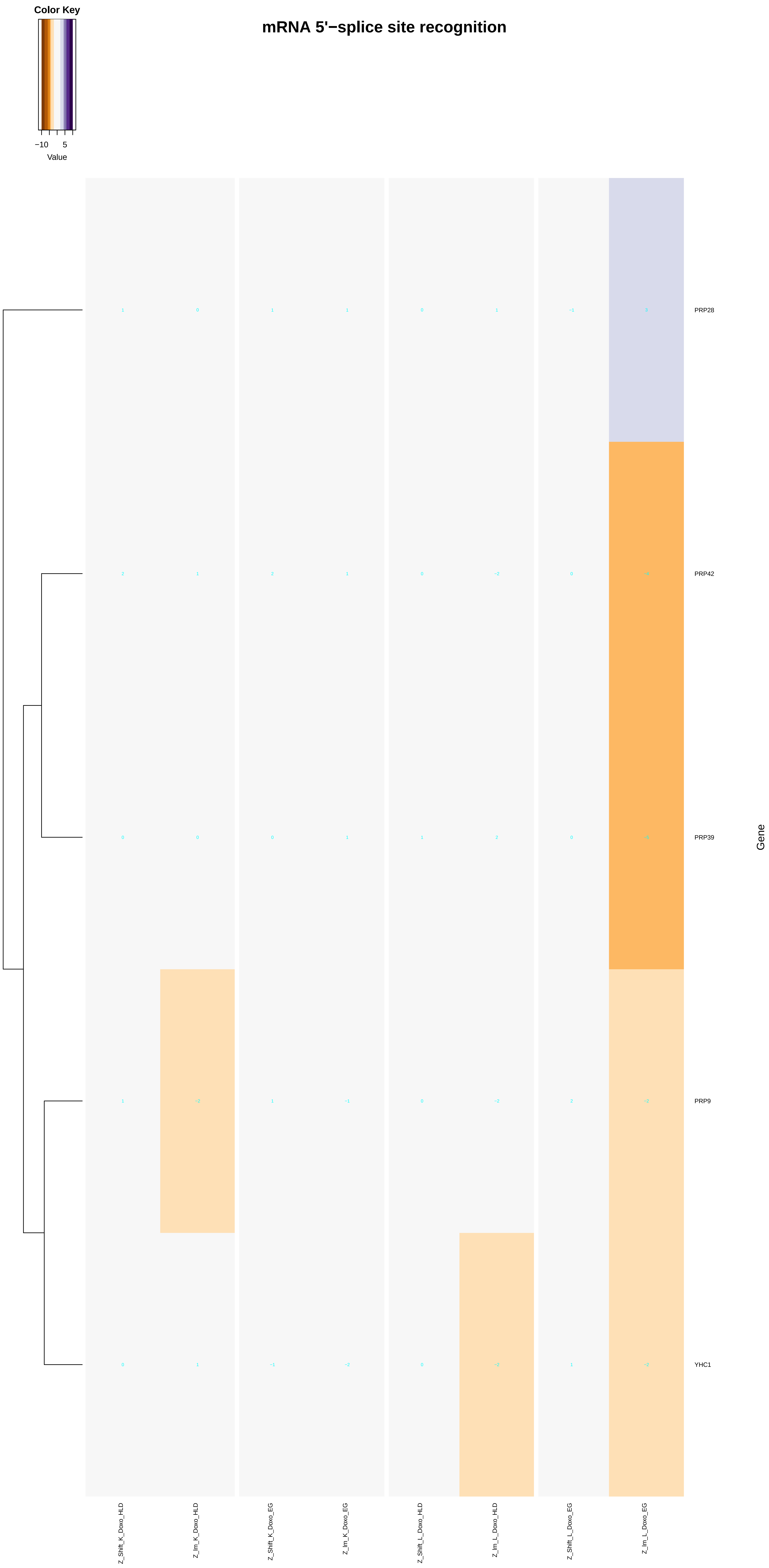
Color Key
−10
5
Value
mRNA 5'−splice site recognition
PRP28
PRP42
PRP39
PRP9
YHC1
Gene
Z_Shift_K_Doxo_HLD
Z_Im_K_Doxo_HLD
Z_Shift_K_Doxo_EG
Z_Im_K_Doxo_EG
Z_Shift_L_Doxo_HLD
Z_Im_L_Doxo_HLD
Z_Shift_L_Doxo_EG
Z_Im_L_Doxo_EG

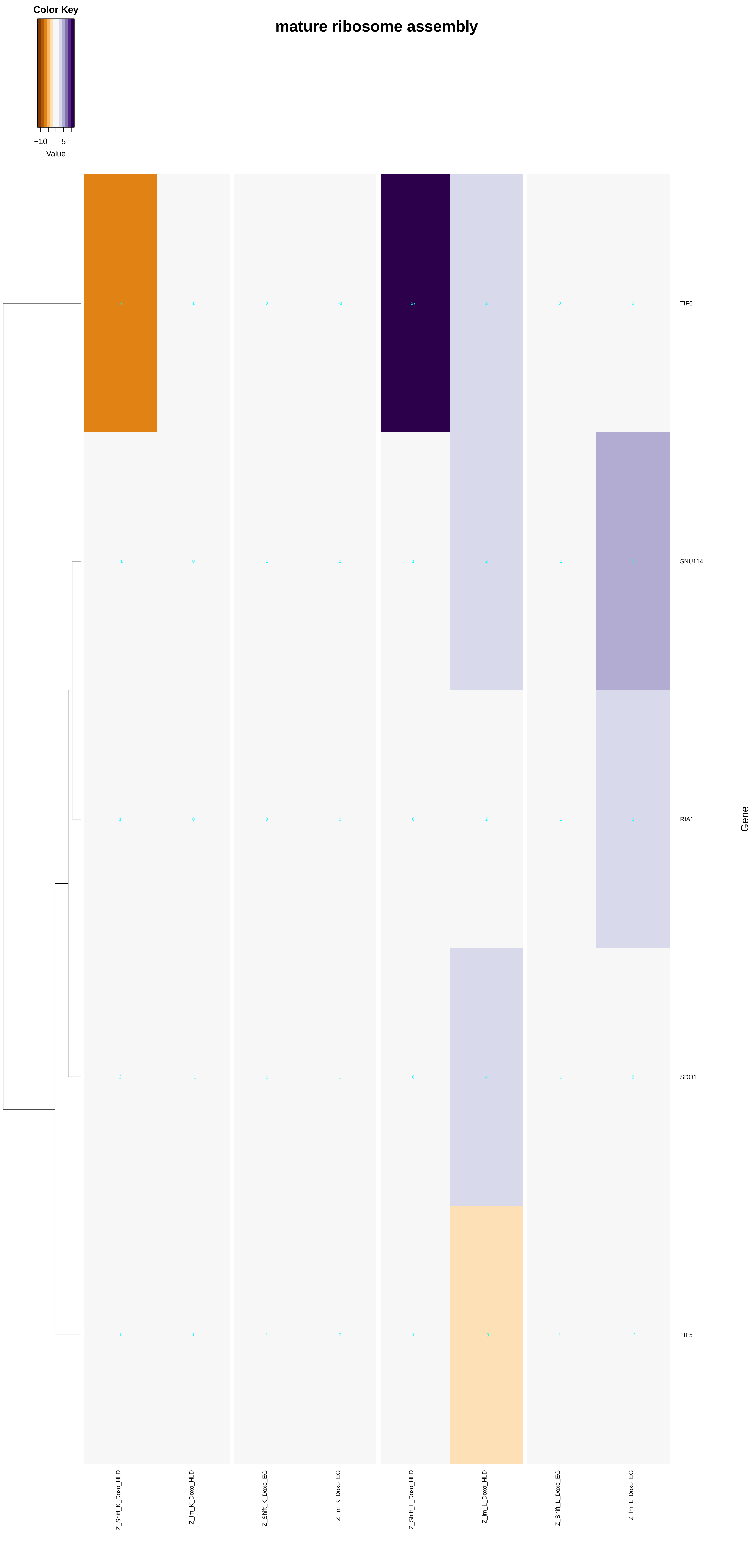

Color Key
−10
5
Value
mature ribosome assembly
TIF6
SNU114
RIA1
SDO1
TIF5
Gene
Z_Shift_K_Doxo_HLD
Z_Im_K_Doxo_HLD
Z_Shift_K_Doxo_EG
Z_Im_K_Doxo_EG
Z_Shift_L_Doxo_HLD
Z_Im_L_Doxo_HLD
Z_Shift_L_Doxo_EG
Z_Im_L_Doxo_EG
